# Microbial communities in the native habitats of *Agaricus sinodeliciosus* from Xinjiang Province revealed by amplicon sequencing

**DOI:** 10.1038/s41598-017-16082-1

**Published:** 2017-11-16

**Authors:** Jiemin Zhou, Xuming Bai, Ruilin Zhao

**Affiliations:** 1 State Key Laboratory of Mycology, Institute of Microbiology, Chinese Academy of Sciences, Beijing, 100101 P.R. China; 2 College of Forestry, Southwest Forestry University, Kunming, 650224 Yunnan Provinve P.R. China; 3 College of Life Sciences, University of Chinese Academy of Sciences, Beijing, 100408 P.R. China

## Abstract

*Agaricus sinodeliciosus* is an edible species described from China and has been successfully cultivated. However, no studies have yet reported the influence factors implicated in the process of fructification. To better know abiotic and biotic factors, physiochemical characteristics and microbial communities were investigated in five different soil samples collected in the native habitats of specimens from northern Xinjiang, southern Xinjiang, and Zhejiang Province, respectively. There are major differences in texture and morphology among different specimens of *A. sinodeliciosus* from Xinjiang Province. *A. sinodeliciosus* from southern Xinjiang was the largest. Concentrations of DOC and TN and C/N ratio are not the main reason for the differences. Microbial communities were analyzed to find out mushroom growth promoting microbes (MGPM), which may lead to the differences. Functional microbes were picked out and can be divided into two categories. Microbes in the first category may belong to MGPM. There may be symbiotic relationships between microbes in the second category and *A. sinodeliciosus*. Certain analyses of microbial communities support the hypothesis that interactions between microbes and mushrooms would be implicated in morphological variation of the collected mushrooms. Redundancy analysis results indicate that high DOC/NH_4_^+^-N ratio and NH_4_^+^-N concentration can improve the yield of *A. sinodeliciosus*.

## Introduction

The applications of mushroom-forming fungus in biotechnological potential play an important role in agriculture, ecology, and human health^1^. In many countries, mushrooms are important dietary food^2–5^, and worldwide yield of edible mushrooms can reach up to about 2.5 million tons every year^1^, most of which are basidiomycetes. It was reported that most of edible basidiomycetes cannot be cultivated under laboratory conditions^1^. However, some species of *Agaricus* belonging to Phylum *Basidiomycete*, have been successfully cultured, such as *A. bisporus* ^6–8^ and *A. subrufescens* Peck^9^.

In Xinjiang Province of China, there are large areas of desert with the primary vegetation types of *Tamarix ramosissima*, *Phragmites australis*, and *Haloxylon ammodendron*. A new species, *A. sinodeliciosus*, was reported by our group^10^, which can grow underground at high salinity and pH with a large and edible fruiting body. This mushroom is sold at high price by locals. Ruleless and excessive picking led to environmental damage and species degradation. The yield of *A. sinodeliciosus* is very low limiting incomes of local farmers. Fortunately, it has been successfully cultivated under laboratory conditions with low yield and long harvesting time. To improve local ecological environment and incomes of local farmers, the yield and harvesting time of *A. sinodeliciosus* should be improved and shortened, respectively.

Application of mushroom growth promoting microbes (MGPM) can improve the productivity and reduce the harvesting time. For *A. subrufescens* Peck cultivation, inoculation of *Exiguobacterium* sp., *Microbacterium esteraromaticum*, *Arthrobacter* sp., *Pseudomonas resinovorans*, or *P. alcaliphila* can significantly improve mushroom total fresh matter yield and shorten harvesting time^11^. In another study, MGPM were isolated from casing soil of *A. subrufescens* Peck, which can also reduce harvesting time and improve fresh yield^9^. *P. putida* was found to be the best MGPM to increase the yield of *A*. *bisporus* ^6^. *Pseudomonas* sp. P7014 can enhance mycelial growth and reduce harvesting time of *Pleurotus eryngii* ^12^. However, no studies have yet reported the best MGPM for *A. sinodeliciosus* cultivation.

Soil is a complex system including various microbes^13^, which play an important role in nutrient cycling^14,15^. Some studies have reported interactions between soil microbes and fungi. Microbial community in the native habitats of *Ophiocordyceps sinensis* was studied and 23 phyla were found^16^. Bacterial and archaeal community associated with different lichens were investigated, which showed that *Alphaproteobacteria* was dominant and bacteria may be the component of lichen symbiosis^17^. For cultivation, mushroom compost is necessary. During this process, raw materials are fermented by microbes to decompose organic materials into simple substance^18–20^. Actinomycetes and fungi are found to be the main cellulose decomposers^20,21^. In the wild, the nutrient used for the growth of *A. sinodeliciosus* was degrading biomass decomposed by soil microbes. It is important to understand the interactions between soil microbes and *A. sinodeliciosus*. However, no studies have yet reported the microbial community in the native habitats of *A. sinodeliciosus*.

To better understand the interactions between soil microbes and *A. sinodeliciosus* and find out some beneficial microbes which may improve the productivity and reduce the harvesting time of *A. sinodeliciosus*, microbial communities were analyzed.

## Results

### Physiochemical characteristics of soil samples in the native habitats of different specimens of *A. sinodeliciosus*

**Figure 1:**
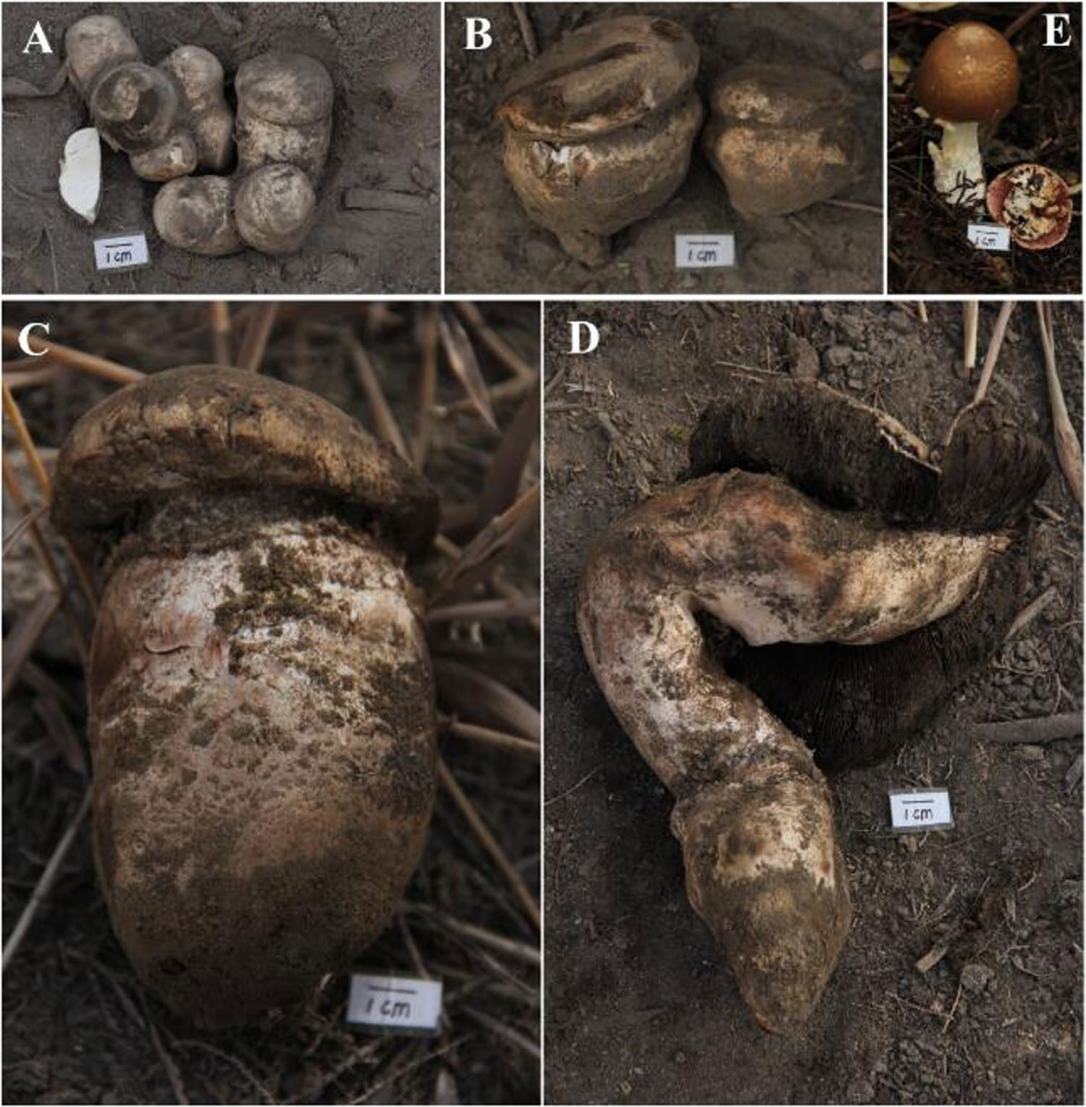
Macrocharacters of different specimens. (**A,B,C,D** and **E**) represent ZRL20152585, ZRL20152589, ZRL20152590, ZRL20152591, and ZRL20151244, respectively. Bar = 1 cm.

**Figure 2:**
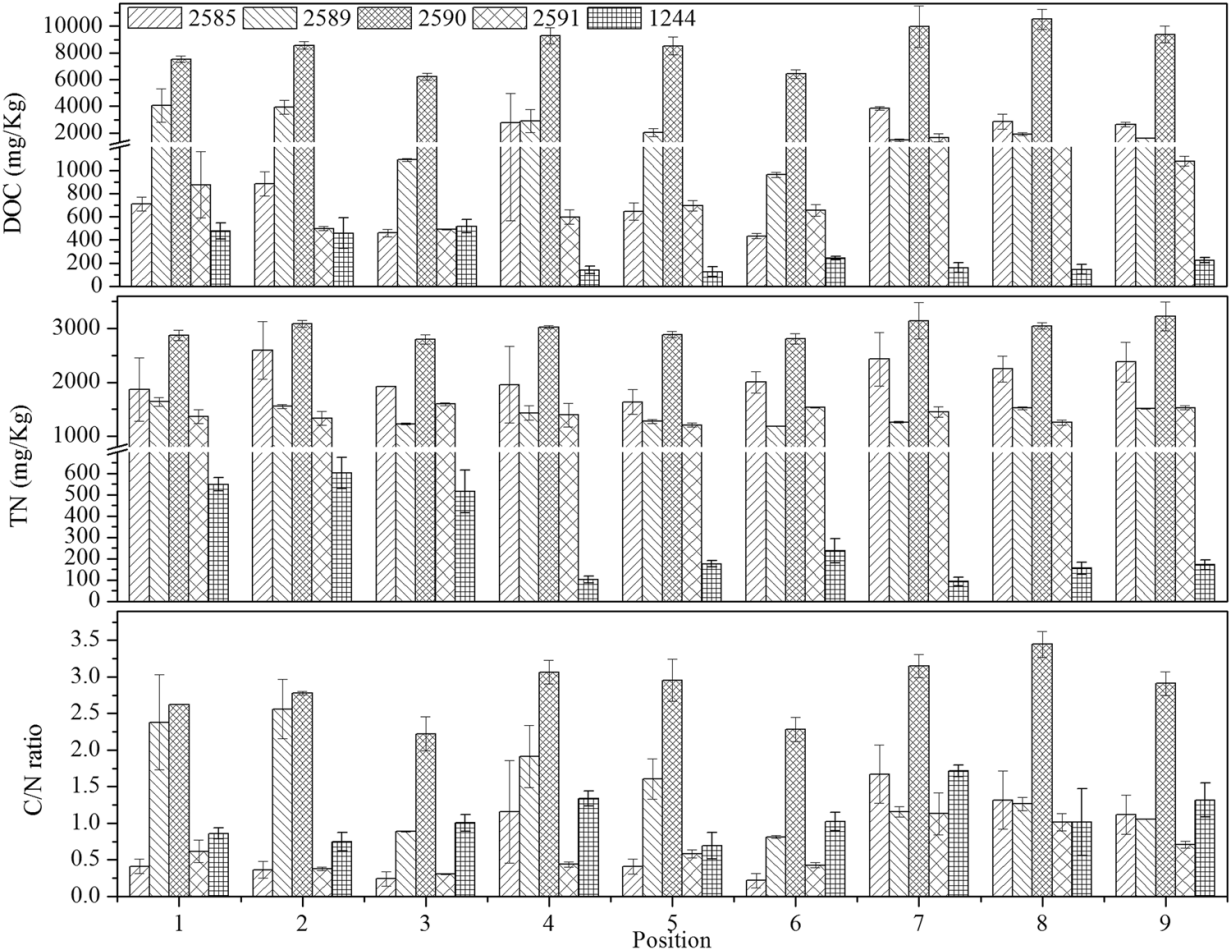
DOC concentration, TN concentration, and C/N ratio in different soil samples. C/N ratio was calculated, dividing DOC by TN.

Major differences in texture and morphology among different specimens of *A. sinodeliciosus* were found. There are many factors linked to mushrooms development^4^. In this study, factors at levels of nutritional state of mushrooms and microbial compositions in the native habitats were investigated. The specimens from Xinjiang Province were about to form cap and the fruiting body cannot be obviously bigger any more. The specimens from southern Xinjiang were obviously larger than those from northern Xinjiang (Fig. 1). Physiochemical characteristics of soil samples were measured (Fig. 2). DOC and TN concentrations of soil samples from Xinjiang Province were higher than those from Zhejiang Province. Concentrations of DOC and TN and C/N ratio of the soil samples in the native habitats of specimen ZRL20152590 were the highest. However, for ZRL20152591 which was also collected from southern Xinjiang, concentrations of DOC and TN and C/N ratio were relatively lower. C/N ratio of soil samples of specimen ZRL20151244 from Zhejiang Province was higher than that of ZRL20152591. These results indicate that concentrations of DOC and TN and C/N ratio are not the main reason for the differences. It was assumed that there may be some MGPM in the native habitats of *A. sinodeliciosus* from southern Xinjiang, which may lead to the differences. Therefore, microbial communities in the native habitats of different specimens were analyzed.

### Richness and diversity of bacterial and fungal communities in different samples

Due to many failures of PCR amplifications, fungal communities of soil samples in the native habitats of specimen ZRL20152590 was not successfully investigated. A total of 4438934 paired-end reads for bacterial communities and 12190961 paired-end reads for fungal communities were produced. After filtering, 2498745 and 1983159 clean tags were obtained, respectively. These clean tags were assigned to 26140 and 5325 OTUs at a 97% similarity, respectively. However, most of rarefaction curves cannot reach saturation (Supplementary Fig. 1), which means that further sequencing is valuable to detect more species. For bacterial communities, the OTU number (Supplementary Table 1) in A06 (838) was the largest among the 45 samples. For fungal communities, the OTU number in D07 (223) was the largest among the 36 samples.

To better understand the differences among the communities, it is important to calculate the richness, evenness, and diversity^22^. Community richness can be demonstrated by Chao1 and ACE^23^. Simpson and Shannon diversity index were used to show community diversity, which demonstrate not only the species richness but the evenness of the species^24–26^. The patterns of Chao1 and ACE were very similar to the OTU numbers^27^. For bacterial communities, on the basis of OTU number, Chao 1 and ACE (Supplementary Table 1), soil sample A had the richest diversity, followed by soil sample D, whereas soil sample E showed the least richness. The Shannon diversity indices of soil sample A and D were higher than those of other specimens. The results of Simpson index were in contrast to those of Shannon diversity index. For fungal communities, soil samples A, D, and E had higher richness and diversity.

### Comparative analysis of bacterial and fungal communities

**Figure 3:**
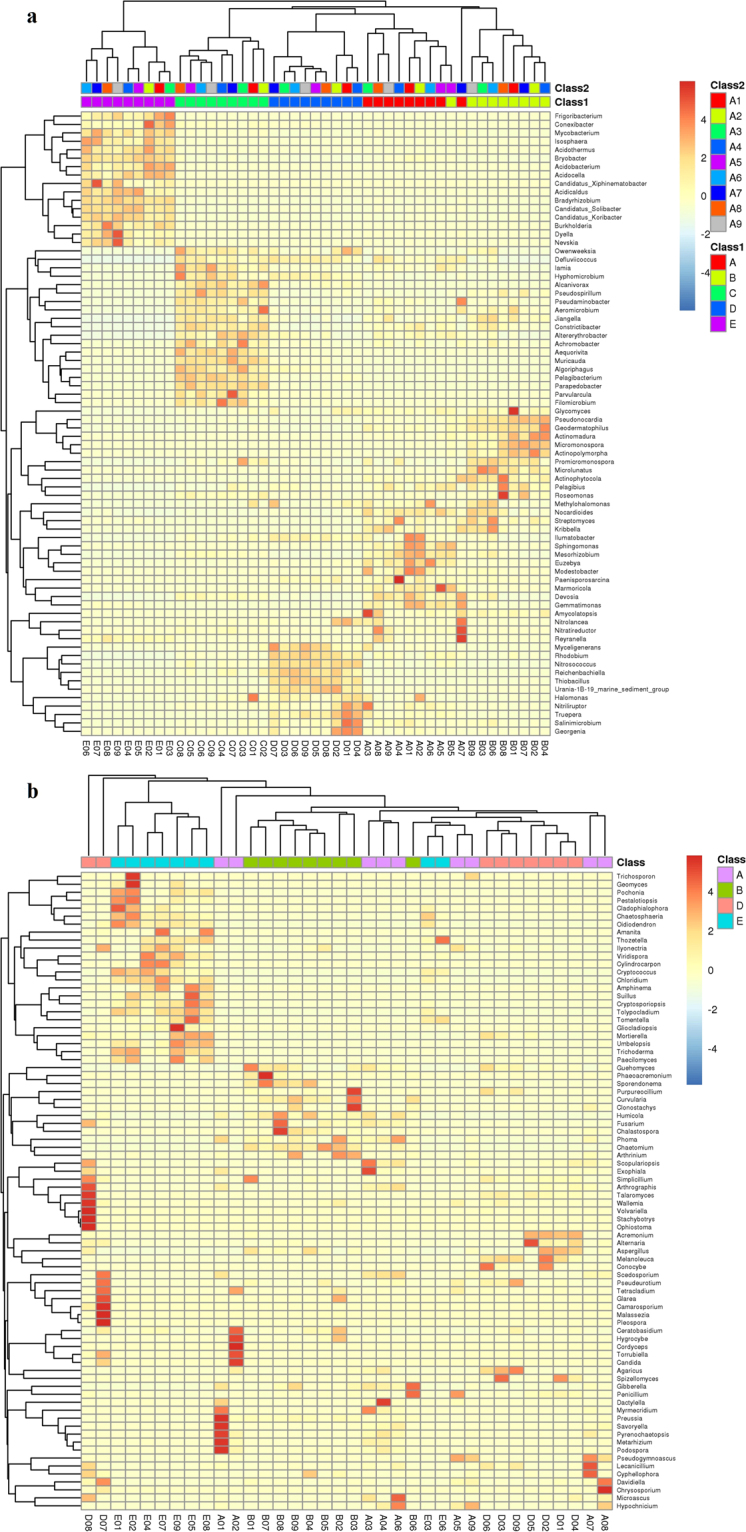
Hierarchical cluster analysis based on 16 S rRNA (**a**) and ITS (**b**) paired-end sequencing. The *Y*-axis is the clustering of the most abundant OTUs (97% similarity) in reads. The *X*-axis is the clustering of different soil samples.

**Figure 4:**
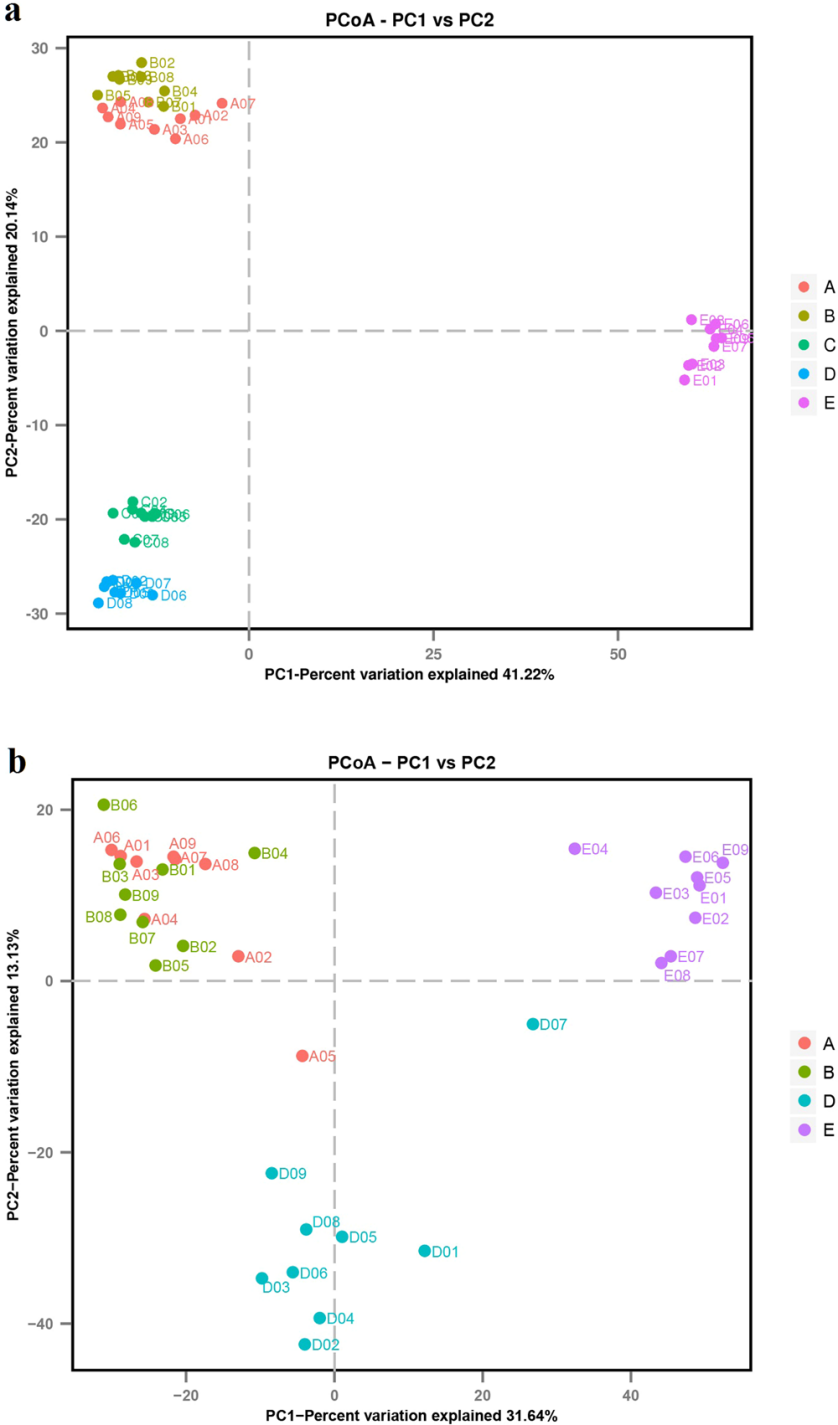
Principal coordinates analysis (PCoA) based on 16 S rRNA (**a**) and ITS (**b**) paired-end sequencing. (A–E) represent the soil samples from specimens of ZRL20152585, ZRL20152589, ZRL20152590, ZRL20152591, and ZRL20151244, respectively.

Hierarchical cluster analysis of communities at genus level was used to demonstrate the different compositions of the microbial community structures (Fig. 3). For bacterial communities, the A-D group was separated from B, C, and E group. For fungal communities, in general, the A-D group was separated from B and E group. These results indicate that there are obvious differences in bacterial and fungal communities among the different samples. Based on considerations of differences in territory among different sampling sites, which can cause the differences in microbial community, there was a hypothesis that A-B, C-D, and E should be clustered together, respectively. Principal Coordinate Analysis (PCoA) was calculated (Fig. 4) and previous studies showed that the results from hierarchical cluster analysis were supported by PCoA^24,25,28^. In this study, for bacterial communities, A-B and C-D were clustered together, respectively and were well separated from E. For fungal communities, A and B were clustered and were well separated from D and E. These results were consistent with the hypothesis.

**Figure 5:**
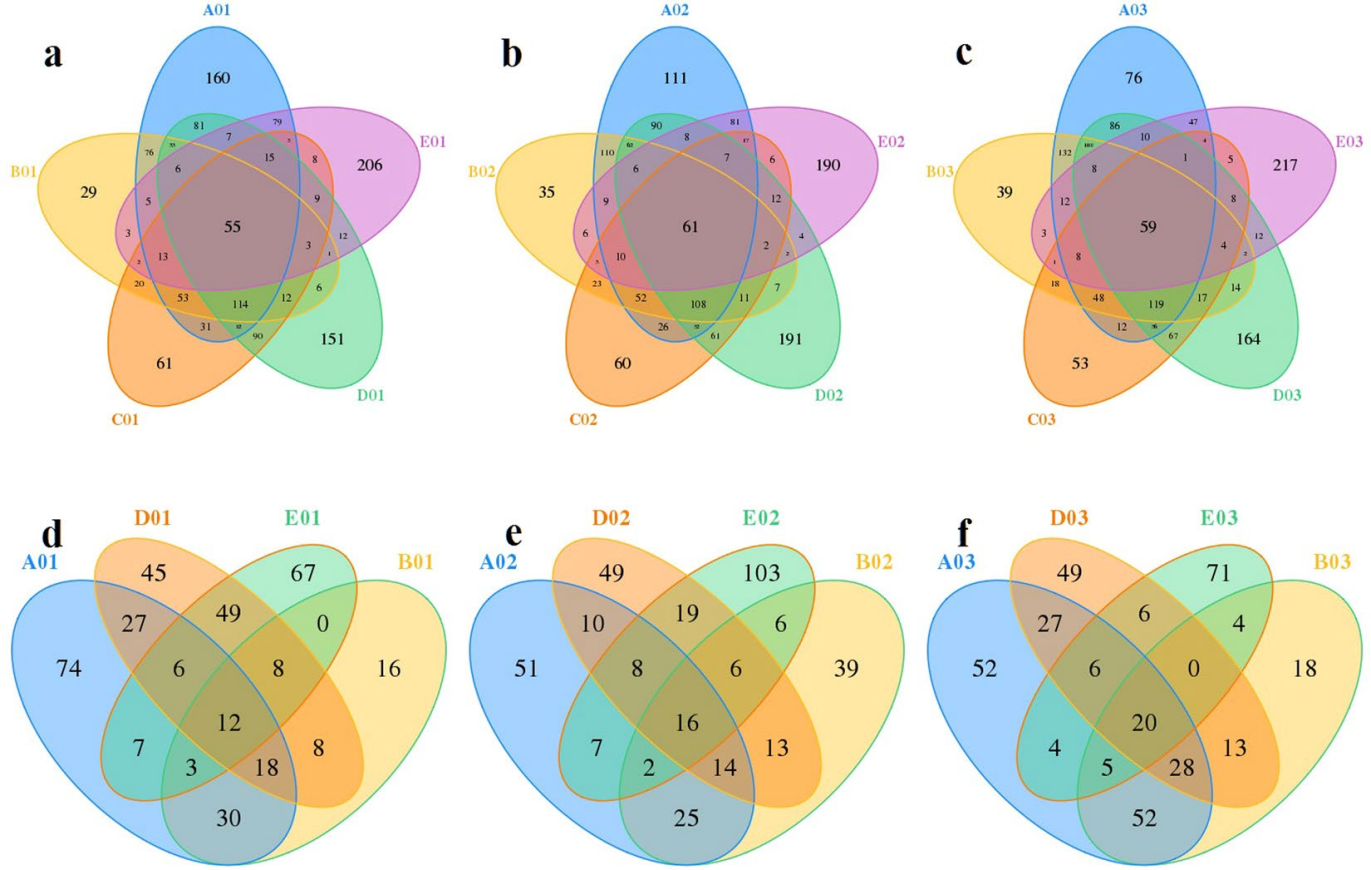
Overlap of the different bacterial (**a**–**c**) and fungal (**d**–**f**) communities. (1–3) represent the topsoil samples from different specimens

**Figure 6:**
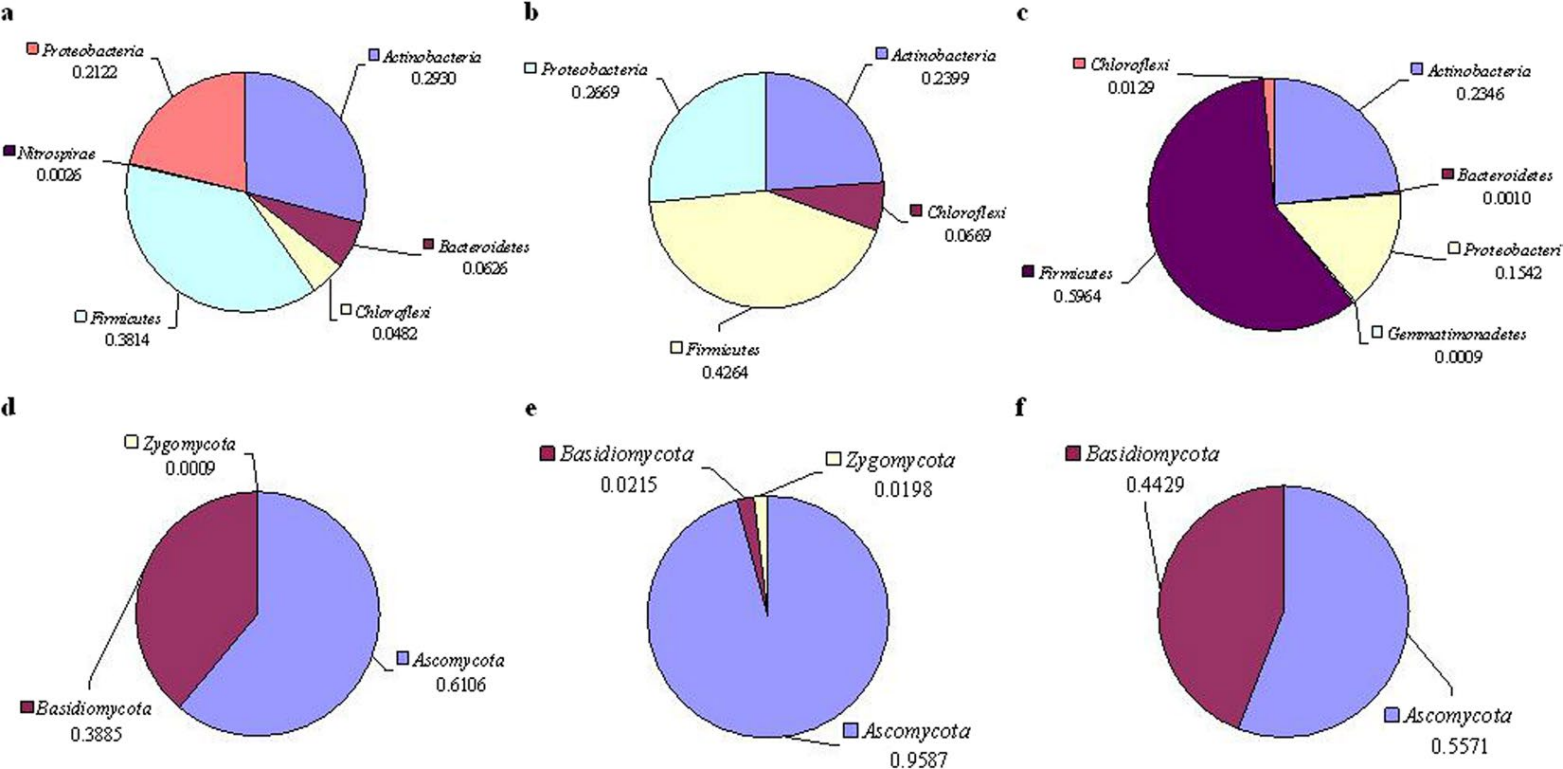
Taxonomic identities of the shared OTUs in Fig. 5 at phylum level. (**a**–**c**) represent bacterial compositions of (1–3), respectively. (**d**–**f**) represent fungal compositions of (1–3), respectively.

Unique and shared OTUs in the same sampling sites (01, 02, and 03) of topsoil from different specimens were summarized (Fig. 5). For bacterial communities, there were three main phyla in the shared OTUs: *Actinobacteria*, *Firmicutes*, and *Proteobacteria*, and *Firmicutes* were highly enriched with relative abundance of 59.64% in the shared OTUs of A03B03C03D03E03 (origin of mushroom) (Fig. 6). Some microbes belonging to *Actinobacteria*, Gram-negative *Proteobacteria* or Gram-positive *Firmicutes* could promote spore germination and hyphal elongation of fungi^29–31^. For fungal communities, the main phyla were *Ascomycota* and *Basidiomycota* (Fig. 6). However, the relative abundance of *Basidiomycota* in the shared OTUs of A03B03D03E03 was very high. This was due to the contamination of mushroom mycelium. If this reason was taken into account, the dominant phylum was *Ascomycota* in the shared OTUs of A03B03D03E03. Fungal decomposers of cellulose included *Ascomycota* and *Basidiomycota* ^32^, which play an important role in the degradation of cellulose, the main polysaccharide in the soil, and were beneficial to mycelial growth^9^ improving the mushroom productivity^12^. These results indicate that some microbes belonging to *Firmicutes* or *Ascomycota* may have the ability to improve the yield of mushrooms.

### Microbial compositions

To identify the phylogenetic diversity of microbial communities in different soil samples, qualified tags were assigned to phyla, classes, and genera. The phylum level identification of bacterial and fugal communities is illustrated in Supplementary Fig. 2. In total, 28 and 4 identified phyla were observed, respectively. For bacterial communities, a major difference in phylum level identification of bacterial communities between samples ABCD and E was the relative abundance of *Chloroflexi*, which played an important role in carbon cycling^33^. This difference may be attributed to regional divergence and differences in DOC concentration. It was found that the relative abundance of *Bacteroidetes* in samples C and D was higher than that in samples A, B, and E, especially in sample C. There was a hypothesis that bacteria in *Bacteroidetes* belonged to MGPM. To prove this hypothesis, community compositions at genus level should be detailedly summarized. Some isolates belonging to *Actinobacteria*, Gram-negative *Proteobacteria* or Gram-positive *Firmicutes* were MGPM^29–31^. *Proteobacteria*, *Firmicutes*, and *Actinobacteria* were highly enriched in all samples. The total relative abundance of these three phyla ranged from 19.2% (B02) to 97.2% (B04), only four samples of which were under 50%. These results indicate that the presence of these microbes may be beneficial for the growth of mushrooms. For fungal communities, if contamination of mushroom mycelium was taken into account, the dominant phylum was *Ascomycota*. However, *Zygomycota* was highly enriched in some samples D and E, and *Chytridiomycota* was only detected in D01 and D03.

The taxonomic breakdown at class level is shown in Supplementary Fig. 3. 58 bacterial classes and 11 fungal classes were detected. For bacterial communities, an obvious difference was that *Acidobacteria* and *Actinobacteria* were highly enriched in different soil samples of B, respectively. The relative abundances of *Alphaproteobacteria* and *Acidimicrobiia* in samples A, C, and E were much higher than those in sample B. *Flavobacteriia* was detected in samples C and D with high relative abundance. The relative abundance of *Anaerolineae* in sample D was much higher than that in other samples. *Gemmatimonadetes* was hardly detected in sample E. For fungal communities, the obvious difference was that the relative abundances of *Dothideomycetes* and *Sordariomycetes* in samples A, B, C, and D were higher than those in sample E. On the contrary, *Tremellomycetes* was detected in sample E with high relative abundance.

**Figure 7:**
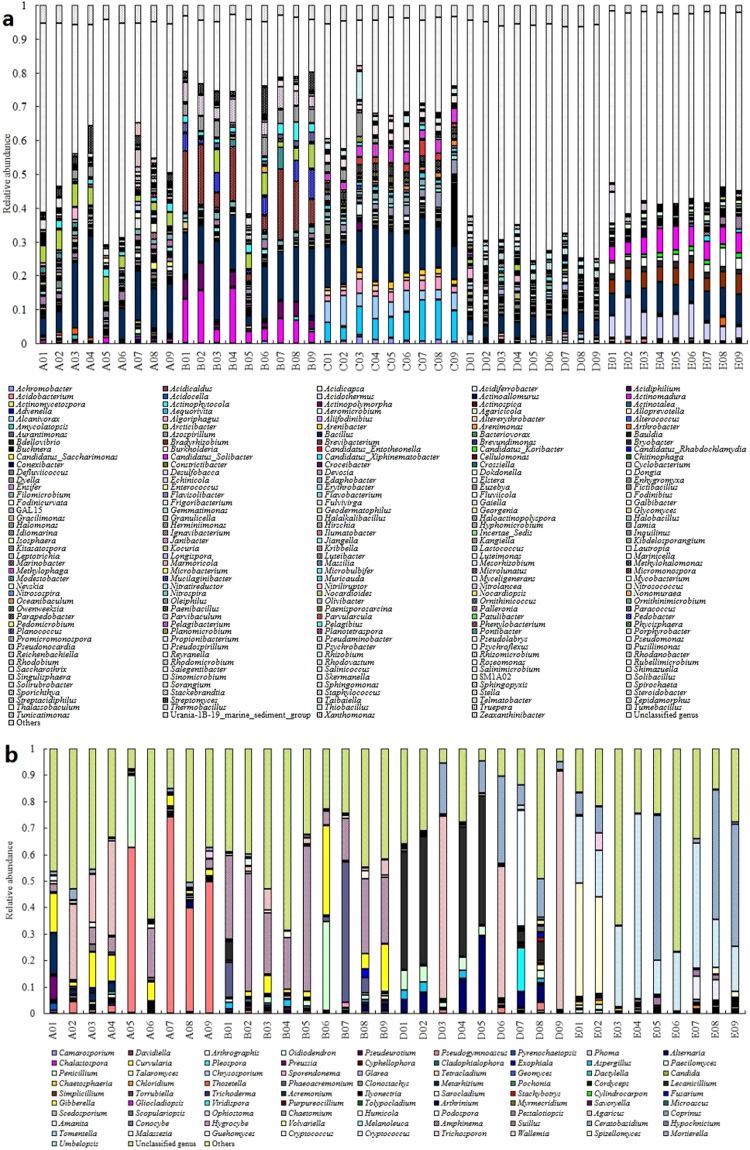
Taxonomic classification of 16 S rRNA (**a**) and ITS (**b**) paired-end sequencing at genus level. Genera making up less than 0.1% of total reads in all communities were classified as “others”.

To prove the hypothesis, community compositions at genus level were detailedly summarized (Fig. 7). In total, 219 bacterial genera and 82 fungal genera were detected. There were great differences between ABCD and E. A lot of clean tags in each sample were not classified at genus level, especially in samples D and E.

## Discussions

Community evenness was found to play an important role in resisting environmental stress^26^, which can be demonstrated by Shannon diversity index. Soil samples from Xinjiang Province were characterized by high salinity and pH. However, for bacterial and fungal communities, the Shannon diversity indices of soil sample E from Zhejiang Province were higher than those of soil sample B and C. Soil in Zhejiang Province was characterized by low salinity and pH. The relatively lower pH may lead to the higher values of Shannon diversity indices of soil sample E.

To find out functional microbes which were beneficial for the growth of *A. sinodeliciosus*, some special microbes were picked out. These special microbes can be divided into two categories. The first category is microbes highly enriched in the topsoil of sample C or D and hardly detected in other samples (Supplementary Table 2). The second category is microbes only detected in the soil of B, C, and D, and not detected in the soil of A and E (Supplementary Table 3). It was assumed that some microbes in the first category belong to MGPM, which have the ability to improve the productivity and reduce the harvesting time^6,9,11,12^. The microbes can be divided into five kinds. The first one was petroleum degraders. *Alcanivorax* was detected in sample C with high relative abundance (Fig. 7), which was considered as obligate hydrocarbonoclastic bacteria and took an important role in biological removal of petroleum hydrocarbons from petroleum-contaminated marine environments^34–36^. *Altererythrobacter* was an important petroleum-aromatic degrader in marine environments^37^, which was also detected in sample C. These two genera were also detected in other samples from Xinjiang Province and not detected in sample E. Based on these results, there was an assumption that long long ago, the desert of Xinjiang Province may be a lake with high salinity and pH, which was polluted by petroleum.

The second one was microbes associated with nitrogen metabolism. *Achromobacter* highly enriched in sample C03 can grow anaerobically with KNO_3_^38^. *Parapedobacter*, only detected in sample C and highly enriched in sample C03, can reduce nitrate into nitrite^39^. *Filomicrobium*, also only detected in sample C, was reported to be isolated from oil-polluted saline soil and positive for nitrate reduction activity^40^. The finding of *Filomicrobium* was consistent with the assumption. *Halomonas* can convert nitrate into nitrogen at high salinity and pH^28,41–43^, which was detected in samples A, C, and D, and highly enriched in sample C01. *Thiobacillus* was only detected in sample D, which was able to reduce nitrate and nitrite^44^. The finding of these microbes indicates that there is nitrate in the soil of Xinjiang, and data of ion chromatograph shows that nitrate concentration was very high in the soil, especially in the topsoil of sample C (Supplementary Table 4). Paired-end sequencing detected not only denitrifying bacteria but also ammonia-oxidizing bacteria and nitrite-oxidizing bacteria. *Nitrosococcus* detected in sample D was reported to be able to utilize ammonia as energy source and reducing power for growth with nitrite as end product^45^. *Nitrolancea* detected in sample D can use nitrite or formate as energy source and CO_2_ as carbon source^46^. The finding of these microbes indicates that nitrogen cycle existed in soil from sample D.

The third one was microbes associated with sulfur metabolism. *Thiobacillus* only detected in sample D was reported to be able to oxidize sulfide into elemental sulfur and convert nitrate into nitrogen simultaneously^47,48^. Sulfide was the product of sulfate reduction^41,42,49^. *Desulfobacca* also only detected in sample D was acetate-degrading sulfate reducer^50^. Although data of ion chromatograph shows that sulfate concentration was also very high in the soil of Xinjiang Province, bacteria associated with sulfur metabolism was not detected in other samples. It was found that sulfate concentration in the topsoil of sample D was the highest (Supplementary Table 4), which may lead to this difference. In samples A, B, and C, there may be some rare microbes associated with sulfur metabolism, which were not detected under the current sequencing depth.

The fourth one was cellulose decomposers. It was reported that *Owenweeksia* producing oxidase, catalase, and alkaline phosphatase under high salinity conditions cannot use cellulose^51^, which were mainly detected in samples C and D. *Iamia* is positive for oxidase and catalase and can reduce nitrate into N_2_^52^, which was highly enriched in sample C and hardly detected in other samples. *Constrictibacter* and *Algoriphagus*, highly enriched in sample C, can produce acid and alkaline phosphatase, esterase (C4), esterase lipase (C8), and β-glucosidase^53,54^, which plays an important role in cellulose degradation. *Aspergillus* can produce endo-glucanase and β-glucosidase^55^, which was detected in the topsoil of A, B, and D, and was highly enriched in sample D. Cellulose is the main polysaccharide in the soil^32^. Cellulose decomposers play an important role in the degradation of cellulose, which was beneficial to mycelial growth^9^ improving mushroom productivity^12^. It was reported that in the acidic topsoil, cellulolytic bacteria included *Betaproteobacteria*, *Bacteroidetes*, and *Acidobacteria*, and fungal decomposers included *Ascomycota* and *Basidiomycota*, which were represented by *Trichosporon* and *Cryptococcus* ^32^. Topsoil of Xinjiang and Zhejiang Province was alkaline and acidic, respectively. The bacterial decomposer *Constrictibacter* found in the topsoil of Xinjiang Province belongs to *Alphaproteobacteria* ^53^ and *Algoriphagus* belongs to *Bacteroidetes* ^54^, respectively. The fungal decomposers found belong to *Ascomycota* ^56^, which can be used as evidence of result that some microbes belonging to *Ascomycota* may have the ability to improve the yield of mushrooms (Fig. 6). The finding of this study provided some evidence for the hypothesis that bacteria in *Bacteroidetes* belong to MGPM, which was in agreement with the literature^32^. However, *Alphaproteobacteria* was not detected in the literature, which was mainly due to the difference in pH value between the literature and our study. The composition of decomposers in the topsoil of Zhejiang Province was in agreement with the literature.

The fifth one was hormones producers which can secrete bioactive growth regulators^57^. *Promicromonospora* can produce gibberellins promoting plant growth and development^57^. In this study, *Promicromonospora* highly enriched in sample C03 was detected in samples A, B, C, and D and not detected in sample E. This result indicates that *Promicromonospora* may produce hormones promoting mushrooms growth, which was related to the fact that mushrooms from Xinjiang Province were much larger than those from Zhejiang Province and mushrooms from southern Xinjiang was larger than those from northern Xinjiang.

There must be some microbes in the first category belonging to MGPM. It was reported that for *A. bisporus* cultivation, *P. putida* was found to be the best MGPM^6^. 1-octen-3-ol produced by conidia of *Penicillium paneum* can inhibit the germination process^58^, which can be consumed by *P. putida*. Therefore the yield of mushroom was increased^6^. However, the species of MGPM and the promoting mechanism for *A. sinodeliciosus* were still unknown, which need further studies.

The microbes in the second category can be regarded as typical microbes of *A. sinodeliciosus* and there may be symbiotic relationships between the typical microbes and *A. sinodeliciosus*. It was reported that land plants and soil fungi of the phylum *Glomeromycota* can form arbuscular mycorrhizal (AM) symbiosis^59^. Bacteria was reported to be component of the lichen symbiosis^17^. Whether the symbiotic relationships existed or not still need further studies. Microbial community analyses indicate that interactions between functional microbes and mushrooms have something to do with the differences in texture and morphology among different specimens.

**Figure 8:**
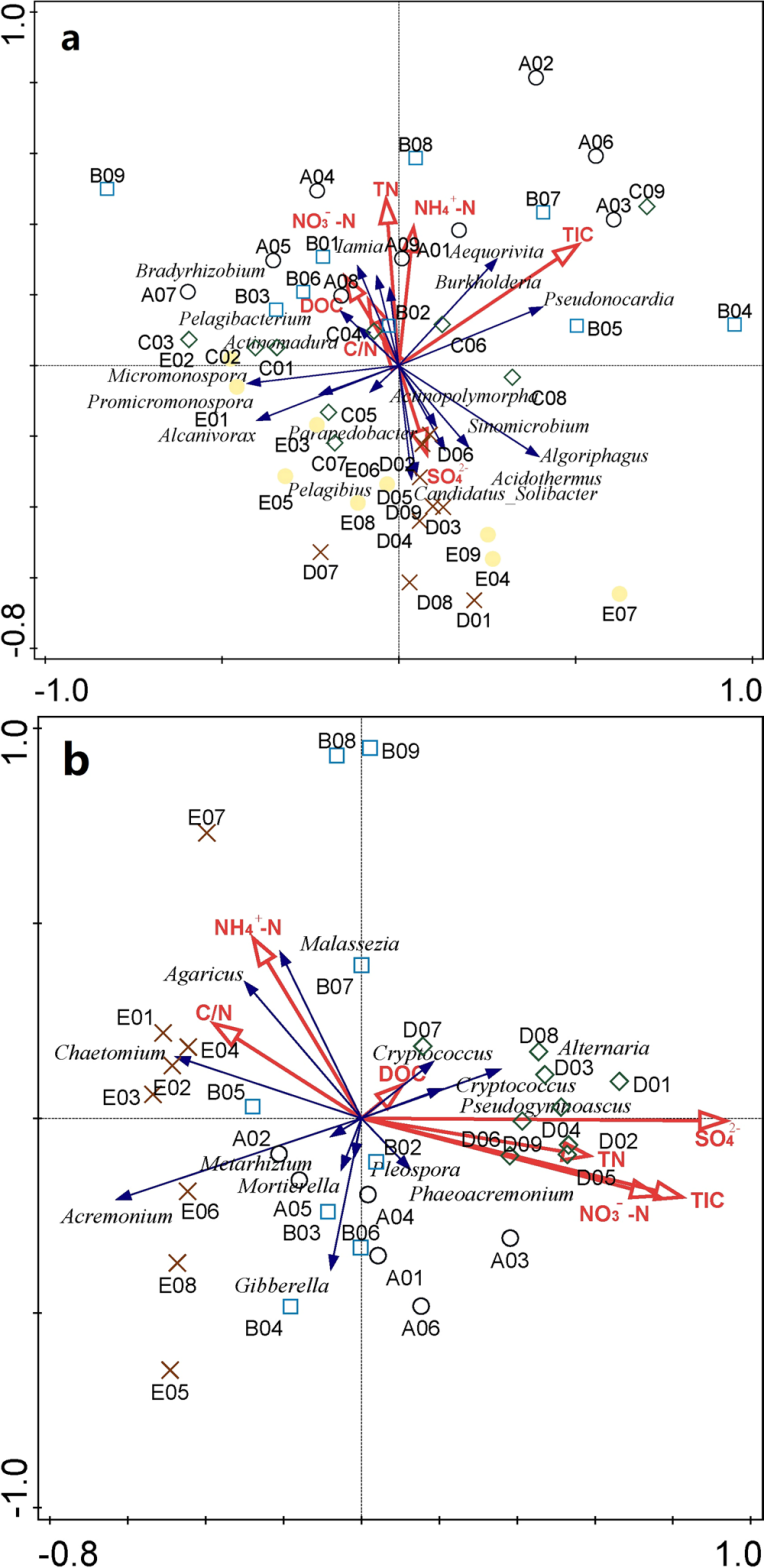
RDA biplots. (**a**) Relationships between bacterial community compositions of samples or bacterial groups and environmental variables; (**b**) Relationships between fungal community compositions of samples or fungal groups and environmental variables.

RDA biplots (Fig. 8) were drawn to reveal the relationships between microbial community compositions of samples or microbial groups and environmental variables. Different environmental variables made great influences. It was found that high C/N ratio, DOC concentration, NO_3_^−^-N concentration, and TN concentration were related to the bacterial communities living in samples C01, C02, and C03. High nutrient concentrations were also related to the bacterial community compositions in the intertidal wetland^60^. However, the bacterial community compositions of samples D01, D02, and D03 also collected from the southern Xinjiang were not related to those high nutrient concentrations, which were related to the high sulfate concentration. This may be due to the high sulfate concentration in the topsoil of sample D, which turned to be main influence factor among the different environmental variables and this may be able to explain the relationship between high NO_3_^−^-N concentration and the bacterial community compositions of samples C01, C02, and C03, too. Relative abundances of *Iamia*, *Aequorivita*, and *Pelagibacterium* belonging to the functional microbes were associated with high nutrient concentrations, and *Algoriphagus* and *Parapedobacter* were associated with high sulfate concentration. High concentrations of sulfate, NO_3_^−^-N, DOC, TIC, and TN were related to the fungal communities living in samples D01, D02, and D03. AM fungal community compositions were also related to soil NO_3_^−^-N content^61^. Relative abundance of *Alternaria* was associated with high concentrations of sulfate, NO_3_^−^-N, DOC, TIC, and TN, which were negatively correlated with relative abundances of *Acremonium* and *Mortierella*. It was interesting that relative abundance of *Agaricus* was associated with high C/N ratio and NH_4_^+^-N concentration, which was inconsistent with the previous results that concentrations of DOC and TN and C/N ratio are not the main reason for the differences in texture and morphology among different specimens. This was due to the fact that for RDA analysis, *Agaricus* was in the form of mycelium not fruiting body. High C/N ratio and NH_4_^+^-N concentration can enhance the mycelial growth of *Agaricus*. According to the determination of NO_3_^−^-N and NH_4_^+^-N (Supplementary Table 4), TN mainly existed in the form of NO_3_^−^-N, which was negatively correlated with relative abundances of *Agaricus*. Reducing NO_3_^−^-N concentration in soil can improve the C/N ratio and thereby enhance the mycelial growth of *Agaricus*. The ratios of DOC to NH_4_^+^-N in the topsoil of *A. sinodeliciosus* were much higher than these in the topsoil of *A. padanus* and *A. planipileus*, especially in the topsoil of specimen C. This result indicates that high DOC/NH_4_^+^-N ratio and NH_4_^+^-N concentration can improve the yield of *A. sinodeliciosus*. RDA analysis can be guidance for the cultivation of *A. sinodeliciosus*.

## Methods

### Study site and sampling

Five different mushroom specimens were collected from northern Xinjiang (ZRL20152585 and ZRL20152589), southern Xinjiang (ZRL20152590 and ZRL20152591), and Zhejiang Province (ZRL20151244) of China, respectively. As shown in Supplementary Fig. 4, soil samples of different horizontal (0, 10 cm, and 20 cm) and vertical (0–5 cm, 5–10 cm, and 10–15 cm) directions were symmetrically collected from the native habitats of different specimens. In total, 27 soil samples were collected from native habitats of each specimens and a total of 135 soil samples were obtained, which were stored at −20 °C in a car refrigerator. To identify mushroom species, DNA was extracted form specimen, and PCR reactions and sequencing were performed using primers ITS1F and ITS4B. Phylogeny of five specimens based on ITS sequences was shown in Supplementary Fig. 5. Specimen ZRL20152585 was clustered with *A. padanus*, and specimens ZRL20152589, ZRL20152590, and ZRL20152591 were clustered with *A. sinodeliciosus*. Specimen ZRL20151244 was clustered with *A. planipileus*. To find out MGPM and typical microbes for *A. sinodeliciosus*, it is necessary to investigate the microbial communities between *A. sinodeliciosus* and other species. In this study, soil samples in the native habitats of specimens ZRL20152585 and ZRL20151244 were used as territorial and interspecific control, ZRL20152589 as territorial and intraspecific control. After being transported to laboratory, soil samples were kept at −80 °C before DNA extraction.

### Analytical methods

Concentrations of sulfate and NO_3_^−^-N were quantified according to previous studies^41,42,49^. Dissolved organic carbon (DOC) and total soluble nitrogen (TN) were measured using a TOC/TNb analyzer (Elementar vario TOC, Elementar Co., Germany)^62^. C/N ratio was calculated, dividing DOC by TN. NH_4_^+^-N was measured by indo phenol blue method^63,64^.

### DNA extraction

Before DNA extraction, soil samples were treated using a 2 mm sieve to remove stone, plant roots, and tissues^16^. Total DNA was extracted from 0.25 g (wet weight) of soil sample using a PowerSoil DNA kit (MoBio Laboratories, CA, USA) following the protocol of manufacturer^28,41^. At the same time, DNA extraction of 135 soil samples was separately performed, and DNA solution from symmetric locations of each specimen was pooled. Finally, for each specimen, 9 DNA solution was obtained (Supplementary Table 5), which was used for sequencing.

### Amplicon sequencing

To determine the diversity and structure of microbial communities, the protocol as previously described was used^65^. PCR amplifications were performed with different primers. For bacterial communities, the primers were 338 F (5′-ACTCCTACGGGAGGCAGCA-3′) and 806 R (5′-GGACTACHVGGGTWTCTAAT-3′)^66^. For fungal communities, the primers were ITS1F (5′-CTTGGTCATTTAGAGGAAGTAA-3′) and ITS2 (5′-GCTGCGTTCTTCATCGATGC-3′)^67^. The primer contains an error-correcting barcode unique to each sample. To minimize the impact of potential early round PCR errors, twenty independent PCR products of each sample were quantified using a Qubit 2.0 fluorometer (Invitrogen, Carlsbad, CA) and then mixed accordingly to achieve equal concentration in the final mixture, which was used to construct PCR amplicon libraries. Sequencing was performed on an Illumina HiSeq platform. Raw sequencing data obtained from this study were deposited to the NCBI Sequence Read Archive database with accession no. SRP093673.

### Data analysis

FLASH was used to merge pairs of reads from the original DNA fragments to produce raw tags^68^. To obtain clean tags, raw tags were filtered strictly according to previous study^69^. First, QIIME (Quantitative Insights Into Microbial Ecology)^70^ quality filters was used to filter raw tags. Then operational taxonomic units (OTUs) were picked using UPARSE pipeline^71^. Sequences were assigned to OTUs at 97% similarities. Alpha diversity and beta diversity were calculated. Venn diagrams were used to describe the similarity and difference among the same sampling sites of topsoil from different specimens. Hierarchical cluster analysis was performed using gplots package of R^24^, and distance algorithm and clustering method were “euclidean” and “complete”, respectively. For PCoA of bacterial and fungal community, distance algorithm was “binary jaccard”. In order to reveal the relationships between microbial community compositions of samples or microbial groups and environmental variables, redundancy analysis (RDA) was performed using CANOCO^72^.

## Electronic supplementary material

Supplementary Information
