# Supplementary material for: Microbial communities in the native habitats of Agaricus sinodeliciosus from Xinjiang Province revealed by amplicon sequencing
Source: Sci Rep. 2017 Nov 16;7:15719. doi: 10.1038/s41598-017-16082-1 (PMC5691072; doi:10.1038/s41598-017-16082-1)
Supplement: Supplementary file 1 — Supplementary Information [file 41598_2017_16082_MOESM1_ESM.doc]

**Microbial communities in the native habitats of *Agaricus sinodeliciosus* from Xinjiang Province revealed by amplicon sequencing**

Jiemin Zhou^a^, Xuming Bai^b^, Ruilin Zhao^a,c,*^

^a^State Key Laboratory of Mycology, Institute of Microbiology, Chinese Academy of Sciences, Beijing 100101, PR China

^b^College of Forestry, Southwest Forestry University, Kunming 650224, Yunnan Provinve, PR China

^c^College of Life Sciences, University of Chinese Academy of Sciences, Beijing 100408, PR China

*Corresponding author: R Zhao. Telephone: +86 10 64806163. E-mail address: zhaorl@im.ac.cn.

**Supplementary Table 1.** Diversity statistics for bacterial and fungal community

| *Position* | *Sample*  *ID* | *Bacterial community* | | | | | | *Fungal community* | | | | | |
| --- | --- | --- | --- | --- | --- | --- | --- | --- | --- | --- | --- | --- | --- |
|  |  | Clean Tags | OTU | ACE | Chao1 | Simpson | Shannon | Clean Tags | OTU | ACE | Chao1 | Simpson | Shannon |
| 2585-1 | A01 | 54643 | 815 | 950.3166 | 992.4783 | 0.008598 | 5.56171 | 55823 | 177 | 205.3835 | 208.0588 | 0.118577 | 2.842317 |
| 2585-2 | A02 | 54638 | 810 | 920.8887 | 935.1375 | 0.008593 | 5.520475 | 56098 | 133 | 205.643 | 283 | 0.130399 | 2.974142 |
| 2585-3 | A03 | 57394 | 766 | 901.906 | 962.2656 | 0.029492 | 4.901896 | 59592 | 194 | 210.032 | 209.7895 | 0.12108 | 2.820677 |
| 2585-4 | A04 | 59469 | 760 | 801.0822 | 810.05 | 0.051213 | 4.610311 | 51674 | 183 | 197.1512 | 200.25 | 0.172713 | 2.562238 |
| 2585-5 | A05 | 48470 | 648 | 756.0875 | 792.8421 | 0.00994 | 5.261186 | 59721 | 99 | 408.2355 | 218 | 0.30746 | 1.656132 |
| 2585-6 | A06 | 53376 | 838 | 947.3458 | 933.3333 | 0.012403 | 5.335719 | 53756 | 193 | 200.6208 | 200.0588 | 0.13207 | 2.772279 |
| 2585-7 | A07 | 54320 | 781 | 889.5381 | 879.8938 | 0.022278 | 4.914839 | 51320 | 98 | 102.7266 | 103.625 | 0.4544 | 1.57232 |
| 2585-8 | A08 | 60393 | 796 | 852.8374 | 885.6792 | 0.013895 | 5.418503 | 56233 | 95 | 99.44472 | 99.66667 | 0.175327 | 2.339695 |
| 2585-9 | A09 | 53920 | 801 | 866.9206 | 890.5082 | 0.019447 | 5.32526 | 52860 | 107 | 112.7201 | 112.5 | 0.201879 | 2.21533 |
| 2589-1 | B01 | 51653 | 431 | 559.7377 | 554.1034 | 0.071719 | 3.383134 | 54162 | 95 | 99.13301 | 102 | 0.223976 | 2.021016 |
| 2589-2 | B02 | 56885 | 507 | 645.6698 | 687.1852 | 0.081028 | 3.340667 | 55098 | 121 | 139.5733 | 134.125 | 0.212744 | 2.566605 |
| 2589-3 | B03 | 53725 | 592 | 674.5774 | 703.1154 | 0.054239 | 4.087528 | 55922 | 140 | 163.2299 | 156.2353 | 0.272954 | 2.00716 |
| 2589-4 | B04 | 57599 | 400 | 529.5322 | 551.9149 | 0.094987 | 3.049848 | 54422 | 143 | 175.4196 | 191 | 0.387126 | 1.728496 |
| 2589-5 | B05 | 56051 | 724 | 783.9267 | 804 | 0.008536 | 5.370306 | 50346 | 158 | 166.9108 | 183.5 | 0.320186 | 1.970261 |
| 2589-6 | B06 | 56577 | 603 | 698.6016 | 731.0377 | 0.034022 | 4.369773 | 57418 | 123 | 147.3408 | 154.9091 | 0.244808 | 1.86199 |
| 2589-7 | B07 | 53997 | 446 | 623.4303 | 605.7971 | 0.083174 | 3.263709 | 56097 | 140 | 156.8237 | 158.4 | 0.336364 | 1.668567 |
| 2589-8 | B08 | 53265 | 454 | 565.7534 | 579.1724 | 0.062346 | 3.537413 | 59337 | 148 | 171.0297 | 168 | 0.124724 | 2.741181 |
| 2589-9 | B09 | 59116 | 560 | 667.9176 | 683 | 0.053651 | 3.87086 | 57030 | 159 | 189.7529 | 188 | 0.165483 | 2.452414 |
| 2590-1 | C01 | 52557 | 573 | 659.9988 | 673.082 | 0.053448 | 4.119453 | - | - | - | - | - | - |
| 2590-2 | C02 | 59233 | 511 | 591.3045 | 612.3333 | 0.047675 | 4.136368 | - | - | - | - | - | - |
| 2590-3 | C03 | 56879 | 460 | 528.6357 | 553.9474 | 0.035567 | 4.127132 | - | - | - | - | - | - |
| 2590-4 | C04 | 50759 | 498 | 590.47 | 639.4286 | 0.039008 | 4.251481 | - | - | - | - | - | - |
| 2590-5 | C05 | 55589 | 457 | 541.701 | 557.0882 | 0.030645 | 4.395085 | - | - | - | - | - | - |
| 2590-6 | C06 | 58025 | 421 | 476.8284 | 476.8 | 0.030681 | 4.291171 | - | - | - | - | - | - |
| 2590-7 | C07 | 56620 | 541 | 625.2608 | 632.1224 | 0.039034 | 4.368339 | - | - | - | - | - | - |
| 2590-8 | C08 | 57251 | 513 | 628.4712 | 610.7759 | 0.033574 | 4.371484 | - | - | - | - | - | - |
| 2590-9 | C09 | 58076 | 440 | 502.645 | 498.1633 | 0.057457 | 3.884209 | - | - | - | - | - | - |
| 2591-1 | D01 | 52352 | 677 | 719.064 | 732.1224 | 0.009624 | 5.420217 | 57619 | 173 | 193.5336 | 192.3333 | 0.215242 | 2.142058 |
| 2591-2 | D02 | 58843 | 684 | 740.5297 | 763 | 0.009317 | 5.442098 | 51130 | 135 | 141.1512 | 139.125 | 0.222204 | 2.10761 |
| 2591-3 | D03 | 60509 | 715 | 773.2123 | 788.3529 | 0.009523 | 5.390385 | 50769 | 149 | 166.2086 | 167.4 | 0.442027 | 1.640322 |
| 2591-4 | D04 | 59899 | 715 | 763.9668 | 777.3077 | 0.011553 | 5.379958 | 59699 | 133 | 150.1509 | 158.3 | 0.205875 | 2.217226 |
| 2591-5 | D05 | 56084 | 650 | 723.8364 | 754.65 | 0.013155 | 5.210069 | 52406 | 106 | 112.9902 | 123.5 | 0.267692 | 1.816223 |
| 2591-6 | D06 | 56669 | 696 | 803.4449 | 792.6667 | 0.011962 | 5.233841 | 55186 | 155 | 173.2545 | 167.0476 | 0.27326 | 2.014951 |
| 2591-7 | D07 | 52347 | 762 | 845.8328 | 865.3621 | 0.009687 | 5.470419 | 53364 | 223 | 272.5968 | 266.0435 | 0.196433 | 2.749772 |
| 2591-8 | D08 | 56293 | 702 | 772.0455 | 776.4167 | 0.011973 | 5.260328 | 54770 | 144 | 210.4911 | 191.25 | 0.055665 | 3.390505 |
| 2591-9 | D09 | 55353 | 675 | 764.1864 | 782 | 0.012785 | 5.208819 | 58575 | 111 | 133.1229 | 149.5 | 0.735971 | 0.873746 |
| 1244-1 | E01 | 58529 | 429 | 638.128 | 558 | 0.021001 | 4.758572 | 50132 | 152 | 178.9865 | 194.2727 | 0.253187 | 2.110638 |
| 1244-2 | E02 | 57275 | 424 | 550.425 | 539.5 | 0.016458 | 4.837628 | 56465 | 167 | 191.834 | 192.8333 | 0.186406 | 2.381045 |
| 1244-3 | E03 | 54819 | 401 | 516.6032 | 508.25 | 0.016796 | 4.831319 | 51105 | 116 | 137.1378 | 133.5 | 0.176497 | 2.228553 |
| 1244-4 | E04 | 51483 | 406 | 446.0223 | 463.6522 | 0.019375 | 4.686064 | 52500 | 196 | 224.6896 | 241.7692 | 0.490241 | 1.740894 |
| 1244-5 | E05 | 51054 | 406 | 431.843 | 451.8824 | 0.015918 | 4.875056 | 59070 | 184 | 190.7502 | 188.7143 | 0.159406 | 2.847281 |
| 1244-6 | E06 | 52356 | 409 | 442.1816 | 468.125 | 0.015428 | 4.900703 | 55002 | 117 | 142.6742 | 148.5 | 0.356563 | 1.639493 |
| 1244-7 | E07 | 53768 | 397 | 422.0598 | 432.6522 | 0.01765 | 4.753223 | 52337 | 183 | 192.3273 | 192.7143 | 0.243829 | 2.459095 |
| 1244-8 | E08 | 55280 | 440 | 515.5145 | 578.3158 | 0.016427 | 4.849565 | 56885 | 207 | 218.5191 | 238.6667 | 0.083785 | 3.188177 |
| 1244-9 | E09 | 55352 | 406 | 441.4513 | 500.2308 | 0.018249 | 4.748706 | 59236 | 168 | 178.0813 | 177.5455 | 0.083697 | 3.091108 |

**Supplementary Table 2. Functional microbes highly enriched in the topsoil of sample C or D and hardly detected in other samples**

|  | **Highly enriched in the topsoil of sample C or D** | | | |
| --- | --- | --- | --- | --- |
| Bacterial | | | | |
|  | *Iamia* | C | *Owenweeksia* | D |
|  | *Alcanivorax* | C | *Nitrolancea* | D |
|  | *Pseudospirillum* | C | *Rhodobium* | D |
|  | *Pseudaminobacter* | C | *Nitrosococcus* | D |
|  | *Aeromicrobium* | C | *Reichenbachiella* | D |
|  | *Constrictibacter* | C | *Thiobacillus* | D |
|  | *Altererythrobacter* | C | Urania-1B-20_marine_sediment_group | D |
|  | *Achromobacter* | C | *Nitriliruptor* | D |
|  | *Aequorivita* | C | *Truepera* | D |
|  | *Muricauda* | C | *Salinimicrobium* | D |
|  | *Algoriphagus* | C | *Georgenia* | D |
|  | *Pelagibacterium* | C |  |  |
|  | *Parapedobacter* | C |  |  |
|  | *Parvularcula* | C |  |  |
|  | *Filomicrobium* | C |  |  |
|  | *Promicromonospora* | C |  |  |
|  | *Halomonas* | C |  |  |
| Fungal | | | | |
|  |  | *Mortierella* | D |  |
|  |  | *Talaromyces* | D |  |
|  |  | *Acremonium* | D |  |
|  |  | *Alternaria* | D |  |
|  |  | *Aspergillus* | D |  |
|  |  | *Melanoleuca* | D |  |
|  |  | *Conocybe* | D |  |
|  |  | *Spizellomyces* | D |  |

**Supplementary Table 3. Functional microbes only detected in the soil of B, C, and D, and not detected in the soil of A and E**

|  | *Bacterial* | | | *Fungal* | | | |
| --- | --- | --- | --- | --- | --- | --- | --- |
|  | *Gracilimonas* | *Owenweeksia* | *Paracoccus* | *Simplicillium* | *Acremonium* | *Purpureocillium* | *Wallemia* |
| B01 | 0 | 0 | 0.028515 | 0.0105 | 0.07152 | 0 | 0 |
| B02 | 0 | 0 | 0.004289 | 0 | 0.001196 | 0 | 0.001965 |
| B03 | 0 | 0.001681 | 0.014911 | 0 | 0 | 0.003452 | 0 |
| B04 | 0 | 0 | 0.009167 | 0.001308 | 0.003686 | 0 | 0 |
| B05 | 0 | 0 | 0.001216 | 0 | 0 | 0 | 0 |
| B06 | 0.001124 | 0.002742 | 0.010859 | 0 | 0.002992 | 0 | 0 |
| B07 | 0 | 0 | 0.013642 | 0 | 0 | 0 | 0 |
| B08 | 0 | 0 | 0.016427 | 0 | 0 | 0 | 0 |
| B09 | 0 | 0.001286 | 0.027339 | 0 | 0.001584 | 0 | 0 |
| C01 | 0.001553 | 0.002972 | 0.002597 | - | - | - | - |
| C02 | 0 | 0.001036 | 0.002567 | - | - | - | - |
| C03 | 0 | 0 | 0.004035 | - | - | - | - |
| C04 | 0 | 0.003172 | 0.002413 | - | - | - | - |
| C05 | 0 | 0.003386 | 0.001299 | - | - | - | - |
| C06 | 0 | 0.002203 | 0 | - | - | - | - |
| C07 | 0 | 0.002844 | 0.001501 | - | - | - | - |
| C08 | 0 | 0.005876 | 0 | - | - | - | - |
| C09 | 0 | 0.002402 | 0 | - | - | - | - |
| D01 | 0.00398 | 0.005566 | 0.001248 | 0.001903 | 0.444852 | 0 | 0 |
| D02 | 0.002282 | 0.002809 | 0.001053 | 0.001757 | 0.484107 | 0 | 0 |
| D03 | 0 | 0 | 0.00509 | 0.00186 | 0.002439 | 0 | 0.001408 |
| D04 | 0.003257 | 0.003953 | 0.001803 | 0.00192 | 0.487982 | 0 | 0 |
| D05 | 0 | 0 | 0 | 0.001491 | 0.486282 | 0 | 0 |
| D06 | 0 | 0 | 0.003097 | 0.003943 | 0.004326 | 0.001374 | 0 |
| D07 | 0 | 0.001991 | 0.003757 | 0.001998 | 0.03975 | 0 | 0.001185 |
| D08 | 0 | 0 | 0.003123 | 0.010513 | 0.069706 | 0 | 0.008939 |
| D09 | 0 | 0 | 0.003288 | 0.000509 | 0.001261 | 0 | 0 |

***Supplementary Table 4. Concentrations of TIC, NO3--N, NH4+-N , and SO42- in different soil samples***

|  | *TIC^a^*  *(mg kg^-1^)* | *NO_3_^-^-N*  *(mg kg^-1^)* | *NH_4_^+^-N*  *(mg kg^-1^)* | *SO_4_^2-^*  *(mg kg^-1^)* |
| --- | --- | --- | --- | --- |
| ZRL20152585-1 | 90.1 ± 21.4 | 1196.5 ± 63.5 | 2.07 ± 0.443 | 6972.6 ± 2620.7 |
| ZRL20152585-2 | 92.7 ± 8.28 | 1336.2 ± 58.2 | 10.8 ± 5.00 | 4596.2 ± 76.9 |
| ZRL20152585-3 | 129.2 ± 9.66 | 1198.7 ± 10.5 | 12.0 ± 0.0142 | 9844.5 ± 36.3 |
| ZRL20152585-4 | 69.7 ± 22.8 | 1155.3 ± 33.3 | 64.5 ± 36.3 | 5902.7 ± 1502.9 |
| ZRL20152585-5 | 55.5 ±12.8 | 1154.0 ± 27.4 | 1.58 ± 0.0434 | 5149.5 ± 726.2 |
| ZRL20152585-6 | 119.6 ± 6.99 | 1214.6 ± 8.46 | 1.60 ± 0.0995 | 6148.7 ± 219.8 |
| ZRL20152585-7 | 6.4 ± 6.43 | 1268.9 ± 66.3 | 4.51 ± 0.701 | 6355.6 ± 162.4 |
| ZRL20152585-8 | 41.1 ± 9.48 | 1240.6 ± 61.5 | 2.66 ± 0.191 | 7734.8 ± 1870.6 |
| ZRL20152585-9 | 54.5 ± 3.96 | 1257.1 ± 7.00 | 2.60 ± 0.0995 | 7804.8 ± 116.1 |
| ZRL20152589-1 | 47.3 ± 13.8 | 1058.1 ± 2.04 | 77.3 ± 5.59 | 3671.1 ± 180.8 |
| ZRL20152589-2 | 48.67 ± 14.9 | 1050.2 ± 0.127 | 63.7 ± 6.04 | 5378.8 ± 317.1 |
| ZRL20152589-3 | 50.7 ± 0.25 | 1077.2 ± 1.34 | 2.96 ± 0.0995 | 3605.9 ± 141.8 |
| ZRL20152589-4 | 64.3 ± 5.09 | 0 | 102.1 ± 12.4 | 4493.6 ± 705.4 |
| ZRL20152589-5 | 44.4 ± 3.94 | 0 | 100.9 ± 4.96 | 3604.7 ± 140.6 |
| ZRL20152589-6 | 62.4 ± 11.6 | 1091.7 ± 1.02 | 1.76 ± 0.0426 | 3382.2 ± 57.5 |
| ZRL20152589-7 | 75.6 ± 6.87 | 0 | 137.0 ± 4.25 | 3370.6 ± 120.7 |
| ZRL20152589-8 | 61.5 ± 6.17 | 0 | 178.4 ± 2.08 | 3807.7 ± 94.2 |
| ZRL20152589-9 | 46.2 ± 0.725 | 1071.6 ± 4.71 | 85.9 ± 0.142 | 3862.0 ± 256.3 |
| ZRL20152590-1 | 32.7 ± 1.3 | 1308.5 ± 29.7 | 4.31 ± 0.256 | 12000.2 ± 881.8 |
| ZRL20152590-2 | 39.6 ± 21.0 | 1366.6 ± 18.2 | 5.21 ± 0.235 | 14042.1 ± 2161.7 |
| ZRL20152590-3 | 17.7 ± 3.69 | 1308.4 ± 0.0450 | 21.4 ± 0.455 | 11835.5 ± 385.1 |
| ZRL20152590-4 | 65.4 ± 34.5 | 1331.8 ± 20.2 | 2.70 ± 0.187 | 13654.4 ± 642.4 |
| ZRL20152590-5 | 44.7 ± 21.2 | 1304.4 ± 17.1 | 3.14 ± 0.330 | 15335.6 ± 1322.1 |
| ZRL20152590-6 | 67.4 ± 9.68 | 1340.6 ± 9.48 | 2.56 ± 0.298 | 12863.9 ± 411.1 |
| ZRL20152590-7 | 73.2 ± 22.9 | 1482.1 ± 71.1 | 6.49 ± 0.480 | 21026.7 ± 2042.4 |
| ZRL20152590-8 | 110.3 ± 2.93 | 1411.6 ± 12.2 | 6.92 ± 1.65 | 18700.1 ± 1581.6 |
| ZRL20152590-9 | 149.2 ± 16.86 | 1524.3 ±6.49 | 2.81 ± 0.0682 | 14853.5 ± 344.9 |
| ZRL20152591-1 | 90.2 ± 25.4 | 1120.1 ± 20.5 | 3.78 ± 0.387 | 21139.9 ± 5210.3 |
| ZRL20152591-2 | 97.4 ± 5.50 | 1114.9 ± 23.6 | 9.26 ± 0.290 | 15090.3 ± 1865.3 |
| ZRL20152591-3 | 87.4 ± 1.28 | 1152.5 ± 13.0 | 1.80 ± 0.0711 | 18612.1 ± 1224.6 |
| ZRL20152591-4 | 86.4 ± 11.5 | 1123.6 ± 36.0 | 2.64 ± 0.478 | 17880.0 ± 3078.9 |
| ZRL20152591-5 | 91.3 ± 1.67 | 1097.9 ± 12.9 | 6.03 ± 0.776 | 14857.8 ± 1608.9 |
| ZRL20152591-6 | 88.0 ± 6.98 | 1161.0 ± 15.1 | 1.58 | 14633.3 ± 1000.7 |
| ZRL20152591-7 | 40.4 ± 17.7 | 1108.5 ± 7.52 | 6.57 ± 1.31 | 15321.3 ± 3290.1 |
| ZRL20152591-8 | 67.1 ± 3.67 | 1098.2 ± 8.76 | 4.40 ± 0.198 | 17370.7 ± 2256.4 |
| ZRL20152591-9 | 79.2 ± 1.83 | 1156.6 ± 4.52 | 3.81 ± 0.0142 | 16915.8 ± 124.9 |
| ZRL20151244-1 | 0 | 0 | 63.7 ± 2.25 | 0 |
| ZRL20151244-2 | 6.23 ± 0.675 | 0 | 69.1 ± 5.14 | 0 |
| ZRL20151244-3 | 0 | 0 | 45.7 ± 3.55 | 0 |
| ZRL20151244-4 | 0 | 0 | 2.44 ± 0.677 | 0 |
| ZRL20151244-5 | 4.70 ± 1.36 | 0 | 13.2 ± 0.526 | 0 |
| ZRL20151244-6 | 7.20 ± 1.63 | 0 | 19.7 ± 0.0142 | 0 |
| ZRL20151244-7 | 0 | 0 | 1.45 ± 0.125 | 0 |
| ZRL20151244-8 | 3.63 ± 1.23 | 0 | 9.98 ± 3.43 | 0 |
| ZRL20151244-9 | 4.05 ± 1.96 | 0 | 8.61 ± 0.0284 | 0 |

^a^Total inorganic carbon

**Supplementary Table 5.** Location and number

| *Location*  *(horizontal, vertical)* | *Number after pooling DNA solution* | **Number used for sequencing** |
| --- | --- | --- |
| (20,0) | ZRL20152585-1 | A01 |
| (10,0) | ZRL20152585-2 | A02 |
| (0,0) | ZRL20152585-3 | A03 |
| (20,5) | ZRL20152585-4 | A04 |
| (10,5) | ZRL20152585-5 | A05 |
| (0,5) | ZRL20152585-6 | A06 |
| (20,10) | ZRL20152585-7 | A07 |
| (10,10) | ZRL20152585-8 | A08 |
| (0,10) | ZRL20152585-9 | A09 |
| (20,0) | ZRL20152589-1 | B01 |
| (10,0) | ZRL20152589-2 | B02 |
| (0,0) | ZRL20152589-3 | B03 |
| (20,5) | ZRL20152589-4 | B04 |
| (10,5) | ZRL20152589-5 | B05 |
| (0,5) | ZRL20152589-6 | B06 |
| (20,10) | ZRL20152589-7 | B07 |
| (10,10) | ZRL20152589-8 | B08 |
| (0,10) | ZRL20152589-9 | B09 |
| (20,0) | ZRL20152590-1 | C01 |
| (10,0) | ZRL20152590-2 | C02 |
| (0,0) | ZRL20152590-3 | C03 |
| (20,5) | ZRL20152590-4 | C04 |
| (10,5) | ZRL20152590-5 | C05 |
| (0,5) | ZRL20152590-6 | C06 |
| (20,10) | ZRL20152590-7 | C07 |
| (10,10) | ZRL20152590-8 | C08 |
| (0,10) | ZRL20152590-9 | C09 |
| (20,0) | ZRL20152591-1 | D01 |
| (10,0) | ZRL20152591-2 | D02 |
| (0,0) | ZRL20152591-3 | D03 |
| (20,5) | ZRL20152591-4 | D04 |
| (10,5) | ZRL20152591-5 | D05 |
| (0,5) | ZRL20152591-6 | D06 |
| (20,10) | ZRL20152591-7 | D07 |
| (10,10) | ZRL20152591-8 | D08 |
| (0,10) | ZRL20152591-9 | D09 |
| (20,0) | ZRL20151244-1 | E01 |
| (10,0) | ZRL20151244-2 | E02 |
| (0,0) | ZRL20151244-3 | E03 |
| (20,5) | ZRL20151244-4 | E04 |
| (10,5) | ZRL20151244-5 | E05 |
| (0,5) | ZRL20151244-6 | E06 |
| (20,10) | ZRL20151244-7 | E07 |
| (10,10) | ZRL20151244-8 | E08 |
| (0,10) | ZRL20151244-9 | E09 |


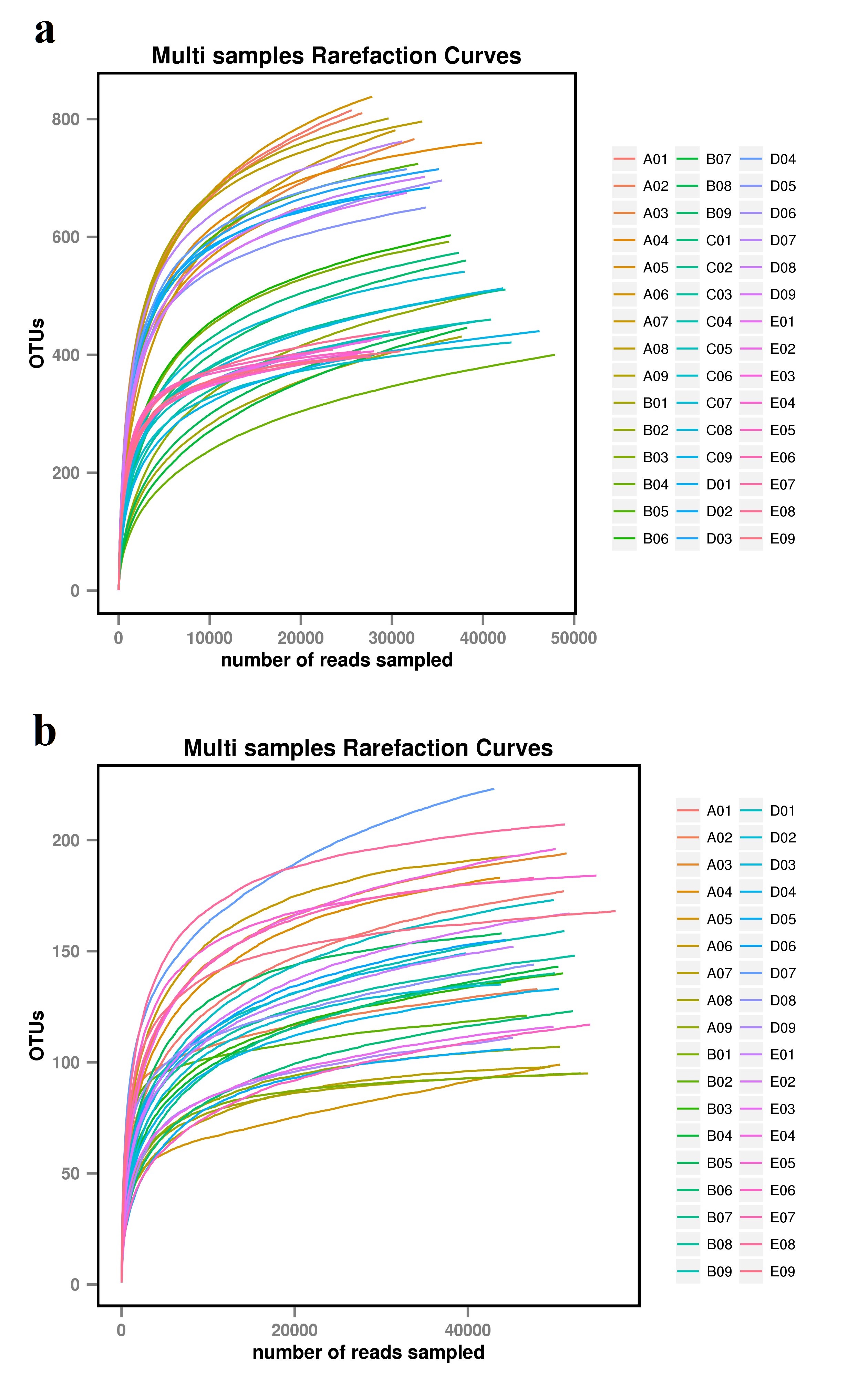


**Supplementary** **Figure 1. Rarefaction curves** **based on 16S rRNA (a) and ITS (b) paired-end sequencing**

| **Bacterial** |
| --- |
|  |
| **Fungal** |
|  |

**Supplementary** **Figure 2.** **Taxonomic classification of 16S rRNA and ITS paired-end sequencing at phylum level.** Phyla making up less than 0.1% of total reads in all communities were classified as “others”.

| **Bacterial** |
| --- |
|  |
| **Fungal** |
|  |

**Supplementary** **Figure 3.** **Taxonomic classification of 16S rRNA and ITS paired-end sequencing at class level.** Classes making up less than 0.1% of total reads in all communities were classified as “others”.


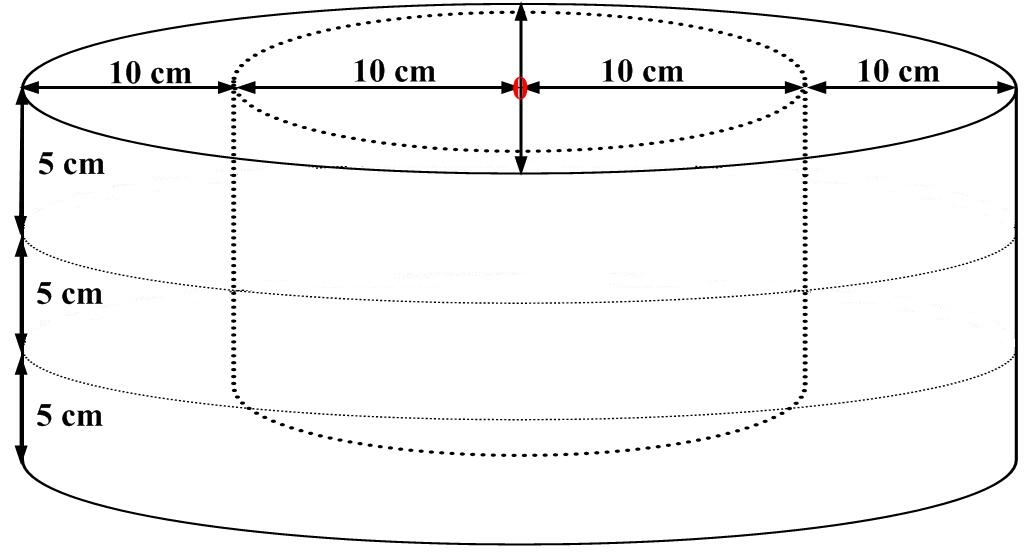


**Supplementary** **Figure 4. Design of sampling location**


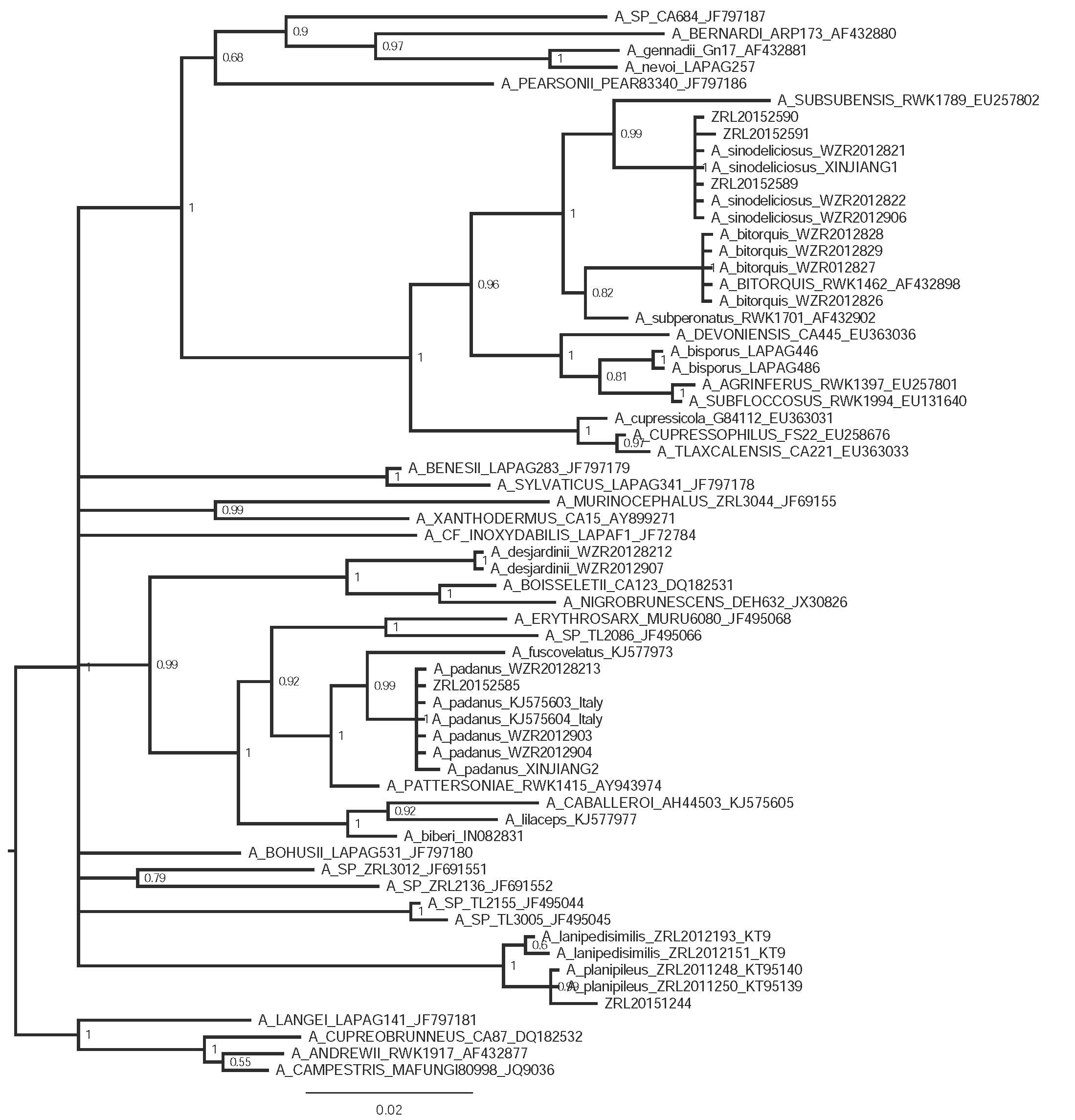


**Supplementary** **Figure 5. Phylogeny of different specimens based on ITS sequences**
